# Prediction of Functionally Important Phospho-Regulatory Events in *Xenopus laevis* Oocytes

**DOI:** 10.1371/journal.pcbi.1004362

**Published:** 2015-08-27

**Authors:** Jeffrey R. Johnson, Silvia D. Santos, Tasha Johnson, Ursula Pieper, Marta Strumillo, Omar Wagih, Andrej Sali, Nevan J. Krogan, Pedro Beltrao

**Affiliations:** 1 Department of Cellular and Molecular Pharmacology, University of California, San Francisco, San Francisco, California, United States of America; 2 Quantitative Cell Biology group, MRC Clinical Sciences Centre, Imperial College, London, United Kingdom; 3 Department of Bioengineering and Therapeutic Sciences, California Institute for Quantitative Biosciences, Byers Hall at Mission Bay, University of California, San Francisco, San Francisco, California, United States of America; 4 Department of Pharmaceutical Chemistry, California Institute for Quantitative Biosciences, Byers Hall at Mission Bay, University of California, San Francisco, San Francisco, California, United States of America; 5 European Molecular Biology Laboratory, Genome Biology Unit, Heidelberg, Germany and European Bioinformatics Institute (EMBL-EBI), Cambridge, United Kingdom; 6 Gladstone Institutes, San Francisco, California, United States of America; 7 iBiMED and Department of Health Sciences, University of Aveiro, Aveiro, Portugal; Indiana University, UNITED STATES

## Abstract

The African clawed frog *Xenopus laevis* is an important model organism for studies in developmental and cell biology, including cell-signaling. However, our knowledge of *X*. *laevis* protein post-translational modifications remains scarce. Here, we used a mass spectrometry-based approach to survey the phosphoproteome of this species, compiling a list of 2636 phosphosites. We used structural information and phosphoproteomic data for 13 other species in order to predict functionally important phospho-regulatory events. We found that the degree of conservation of phosphosites across species is predictive of sites with known molecular function. In addition, we predicted kinase-protein interactions for a set of cell-cycle kinases across all species. The degree of conservation of kinase-protein interactions was found to be predictive of functionally relevant regulatory interactions. Finally, using comparative protein structure models, we find that phosphosites within structured domains tend to be located at positions with high conformational flexibility. Our analysis suggests that a small class of phosphosites occurs in positions that have the potential to regulate protein conformation.

## Author Summary

Proteins can be modified during their life-cycle in order to regulate their function. The addition of a phosphate group is one of the most abundant and well understood protein modifications. Recent technological developments are now allowing us to uncover thousands of phosphorylation sites within proteins in a single experiment. Here we applied these approaches to identify phosphorylation sites in the African clawed frog *Xenopus laevis*. It has been suggested that many such modifications might serve no biological function inside the cell. To address this we have used a comparative approach to identify highly conserved phosphorylation sites and regulatory interactions. In addition we have used 3D structural models to identify a set of phosphorylation sites that might regulate protein conformations.

## Introduction

Protein function can be regulated by post-translational modifications (PTMs) by altering diverse protein properties such as their localization, activity or interactions. Protein phosphorylation is one of the most well studied PTMs with over 50 years of research since the pioneering work of Krebs and Fischer on glycogen phosphorylase [1]. It is estimated that approximately 30% of the human proteome can be phosphorylated and this modification has been shown to play a role in a very broad set of cellular and developmental functions as well as dysregulation in disease [2]. Recent advances in phosphoenrichment procedures and mass spectrometry (MS) technologies have resulted in a tremendous increase in the capacity to identify phosphorylation sites on a large scale [3] and over the past few years over 200.000 phosphorylation sites have been identified across a varied number of species (ptmfunc.com). These studies have highlighted the true extent and complexity of PTM regulation and underscored the need to develop large-scale approaches to study PTM function. The availability of such data for different species has allowed for comparative studies. While a significant level of constraint on phosphorylated residues has been detected [4] a large fraction of phosphosites are not conserved across species [5–12]. Given the high evolutionary turn-over of these modification sites it is plausible that a fraction of these serve no biological purpose in extant species [5,13]. Therefore, it has become important to develop methods to discern the functional relevance of PTMs [14]. For example, the conservation of phosphosites has been used to highlight sites that are more likely to be important. Different kinases have specific preferences for the amino acids in the vicinity of the target phosphorylated residue [15–17]. This local sequence context is often referred to as the kinase target consensus sequence or motif and the conservation of these kinase motifs across orthologous proteins can be used to improve the predictions of kinase regulated sites [18,19]. In parallel to conservation based approaches, computational and experimental methods have been developed to identify phosphosites that are more likely to be functionally important by regulating protein interactions [13,20], protein activities [13], metabolic enzymes [21], or cross-regulate other types of modifications [22–24].

Phosphoproteomic approaches have been applied extensively to several species [7,8,25–28]. However, although *X*. *laevis* is a well-established model organism there has been little previous knowledge of the extent and conservation of its phosphoproteome. To address this we have used a MS approach identify phosphorylation sites in *X*. *laevis* egg extracts. This approach resulted in the identification of 1738 phosphorylation sites. For the subsequent analysis, we combined these sites with sites identified in a previous study [29] resulting in a total of 2636 phosphosites for analysis. Using a compilation of phosphorylation information for 13 other species we identified a small number of highly conserved phosphosites, which were found to be enriched in sites with known molecular functions. In addition we used kinase specificity predictions for kinases involved in cell cycle regulation to predict conserved kinase-protein associations. The degree of conservation of predicted cell cycle-related kinase interactions was correlated with known kinase-protein regulatory interactions. Conserved putative interactions were also enriched in proteins that are phosphoregulated during the cell cycle and in genes that when knocked down cause mitotic phenotypes. In order to study the structural properties of these sites we obtained comparative models for 518 phosphosites. Structural analysis revealed a number of solvent inaccessible phosphosites that likely indicate protein regions that can exist in more accessible conformations. The analysis of these sites suggests that a significant fraction may regulate protein conformation.

## Results

### Conservation and structural coverage of the *X*. *laevis* phosphoproteome

**Fig 1:**
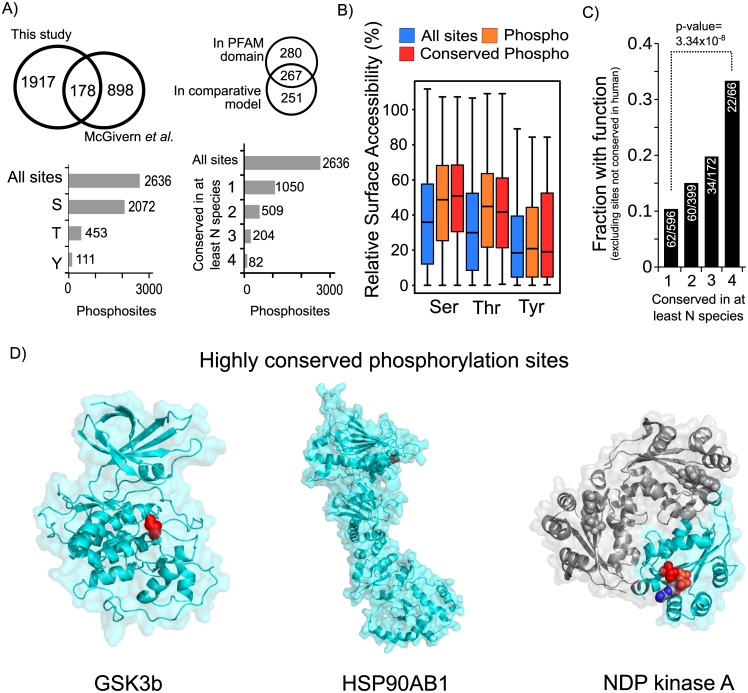
Structural and evolutionary analysis of *X*. *laevis* phosphosites. A) A total of 2636 non-redundant phosphorylation sites were compiled from the sites determined here and those collected from a previous study [29]. We determined the conservation of these 2636 phosphorylation sites across the 13 other species and obtained structural models for 518 of these sites. B) The all-atom residue relative surface accessibility was compared for all phospho-acceptor residues, phosphosites not conserved or conserved in at least one other species with available phosphorylation data. C) The fraction of *X*. *laevis* sites with a known function in human increases with the degree of conservation. *X*. *laevis* sites not conserved in human were excluded from this analysis. D) Example comparative models with highly conserved phosphorylation sites. The phosphorylation site is highlighted in red. For the NDP kinase A, the structure represents the homo-oligomeric complex. One of the subunits is indicated in blue, with the phosphosite position in red and the substrate in the ball-and-stick representation.

*Xenopus laevis* egg extracts were prepared in two cell cycle stages (interphase and mitosis) in order to increase the coverage of phospho-regulatory events. After protein extraction the samples were trypsin digested, subjected to a phosphopeptide enrichment protocol and LC-MS analysis (see Materials and Methods). The spectra were matched to a reference proteome for *X*. *laevis* and phosphosites were identified with a false discovery rate below 1% as estimated by a decoy library search. The localization of the phosphosite acceptor residue with the peptides was scored using the SLIP score [30]. We identified a total of 1738 non-redundant phosphosites in both samples. Using the SLIP scores we estimate the 77% of these phosphosites are well localized within the phosphopeptide (see S2 Table). In addition we have also compiled 1076 sites from a previous study [29] that we were able to map to the same reference proteome. The two sets obtained in this study have a total of 2636 non-redundant sites (S1 Table). The distribution of modified residues is similar to previous phosphoproteomics studies with 2072 (78%) phospho-serines, 453 (17%) phospho-threonine and 111 (4%) phospho-tyrosines (Fig 1A). For most of the analysis we do not require unambiguous localization of the phospho-acceptor residue within the phosphopeptide and used the total set of 2636 phosphosites. To facilitate the re-use of this data we also selected a higher quality subset of 941 phosphosites with high expectation and localization scores (see Methods and S1 Table).

In order to study the structural properties of these phosphosites we obtained comparative models for *X*. *laevis* phosphoproteins. The models were created with ModPipe [31] using templates with at least 25% sequence identity and using established model quality criteria (see Materials and Methods). When different models were available for the same phosphosite-containing region we selected the largest model available. We were able to obtain models for a total of 518 phosphosites of which 267 are contained within known PFAM domain (Fig 1A). An additional 280 are within PFAM domain boundaries for which we could not build a model. Of the total number of sites that are likely to be within globular protein domains, as defined by PFAM, we could model approximately 49%. In addition to structural information we also determined the level of the conservation of the identified phosphosites using a compilation of phosphorylation information for a set of 13 other species obtained from ptmfunc.com (*Saccharomyces cerevisiae*, *Schizosaccharomyces pombe*, *Plasmodium falciparum*, *Toxoplasma gondii*, *Trypanosoma brucei*, *Trypanosoma cruzi*, *Oryza sativa*, A*rabidopsis thaliana*, *Drosophila melanogaster*, *Caenorhabditis elegans*, *Homo sapiens*, *Rattus norvegicus* and *Mus musculus*). The number of predicted orthologs and known phosphosites used for each species is provided in S3 Table. *X*. *laevis* proteins were aligned with putative orthologs in these species and a phosphosite was considered to be conserved in a target species when the aligned peptide region was known to be phosphorylated in that species (see Materials and Methods). Previous studies have noted that regulation by protein phosphorylation can diverge quickly during evolution [5–7,9,10]. In line with these studies we find that only 1050 (39.8%) sites were found to be conserved in one or more species analyzed (Fig 1A). We note that the conservation values of the phosphorylation status are under-estimated due to lack of complete coverage for most phosphoproteomes. We next combined the structural information and the conservation information to study the surface accessibility of the phosphosites. As expected [32,33], phosphosites are on average more likely to have higher all-atom relative surface accessibility than non-modified residues. This is apparent for serines and threonines (Fig 1B, phospho-serines vs. serines p-value = 2.328x10^-9^, phospho-threonines vs. threonine p-value = 3.518x10^-5^ with a two sample Kolmogorov-Smirnov test) but not for tyrosines. Phosphosites conserved in at least one species do not appear to be more surface-exposed than average phosphosites (Fig 1B).

A small fraction of phosphosites was found to be conserved across several species. We observed for example that 82 sites were conserved in 4 or more species (Fig 1A). In order to test the usefulness of this comparative approach we used a list of human sites known to have a molecular function from small-scale studies (from phosphosite.org). We first restricted the analysis to 596 *X*. *laevis* phosphosites conserved in *H*. *sapiens*. We then tested if the level of conservation in additional species beyond human was predictive of a known function in human. While 62 of these 596 (10.4%) *X*. *laevis* sites have a known human function we observed that the fraction of sites with known function increased with the level of conservation of the phosphorylation status (Fig 1C). Of the 66 Xenopus sites that are conserved in human and in at least 3 other species 22 have a known human function (33%). We next predicted protein disorder for *X*. *laevis* proteins using DISOPRED (version 3.1) and repeated this analysis separately for protein regions predicted to be ordered and disordered. In both cases the trend is the same with a stronger enrichment observed for disordered regions (S1 Fig). This large and significant increase suggests that phosphosites conserved across many distantly related species are more likely to be functionally relevant. Increasing coverage of experimentally determined phosphorylation sites across a varied number of species will further facilitate the identification of such highly conserved sites. Examples of highly conserved sites with available homology models are shown in Fig 1D. For example, the phosphorylation of the activation loop region of the GSK3b protein kinase (Fig 1D, left) is one of the most conserved phosphorylation sites across all species. Phosphorylation of the activation loop region of protein kinases is a very well established mechanism to regulate kinase activity. HSP90 proteins are one of the most conserved molecular chaperones involved in the folding of a varied set of client proteins [34]. This chaperone is a highly flexible protein typical forming a homo-dimmer via a c-terminal region [34]. One of the most conserved phosphorylation sites we identified here is in a protein homologous to the human HSP90AB1 (Fig 1D, center). The phosphorylation occurs in the flexible n-terminal region that opens and closes during the ATPase cycle [34]. This position is equivalent to S226 in the human HSP90AB1 that has been previously shown to regulate chaperone activity [35]. Another interesting phosphosite, conserved in 4 other species, is the threonine near the active site of NDP kinase A (Fig 1D, right) which due to the proximity to the substrate is very likely to influence enzyme activity. Given the broad phylogenetic distribution of the species used in this study, these highly conserved sites are expected to be enriched in ancient and functionally important phospho-regulatory modifications. A list of the *X*. *laevis* sites that are phosphorylated in the predicted human ortholog and in at least 4 other species are listed in Table 1, along with annotations on the molecular role from PhosphositePlus (www.phosphosite.org).

**Table 1:** Highly conserved phosphorylation sites. *X*. *laevis* phosphosites conserved in human and at least 4 other species are listed with the corresponding human gene name and phosphosite molecular function, if defined in the Phosphosite plus database.

Protein ID	Phosphosite position(s)	Conserved in N species	Human homolog gene name	Known function
XL_00048253	219	11	GSK3B	
XL_00292621	176	8	CDK7	Induced kinase activity
XL_00213365	38	8	RPL12	
XL_00242882	438	7	RPLP0	
XL_00077149	226	7	HSP90AB1	Induced HSP90 activity
XL_00277348	108	7	EEF1B2	
XL_00157535	251	6	CCT3	
XL_00152649	245; 246; 250	6	RPS6	Regulation of molecular association
XL_00007232	13	6	MCM2	Induced activity
XL_00237045	899; 900; 901	5	GBF1	
XL_00131108	646; 647	5	CLASP1	
XL_00154464	430	5	RPS6KB1	Induced enzymatic activity
XL_00152649	251; 252	5	RPS6	
XL_00232516	101	5	PRKAR2A	
XL_00007232	26	5	MCM2	Induced activity
XL_00007232	25	5	MCM2	Induced activity
XL_00277462	14	5	SEC61B	

### Phosphosites are associated with regions with high conformational flexibility.

**Fig 2:**
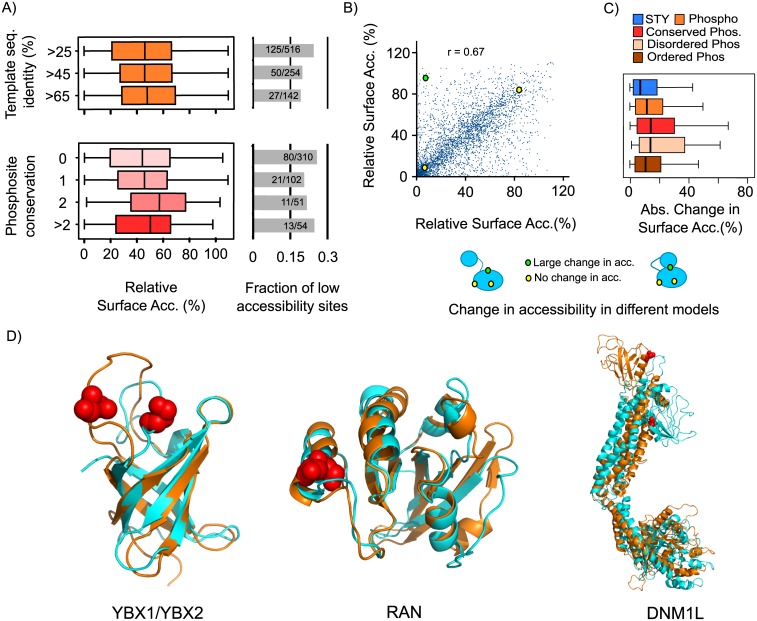
Phosphosites in solvent inaccessible positions may be predictive of conformational flexibility. A) A small fraction of phosphosites (approximately 20%) was observed to be at solvent inaccessible positions (defined here as <20% all-atom RSA). The distribution of phosphosite RSA and the fraction of low accessibility sites do not vary significantly as a function of target-templates sequence identity nor phosphosite conservation. B) For phospho-acceptor residues (Serine, Threonine and Tyrosine) modeled independently based on more than one template structure, we compared the RSA values obtained from different models. These values are highly correlated, although some sites showed large variability in predicted accessibility, potentially indicating regions of conformational flexibility. C) We compared the changes in RSA in different models for phospho-acceptor residues, phosphoryation sites not known to be conserved, those conserved in at least 1 other species, phosphosites predicted by DISOPRED to be in ordered or disordered regions. D) Examples of phosphosites found in positions that show a large change in accessibility in two templates and are poorly accessibility in one of the templates. The phosphosite position is highlighted in red and the models in orange have a higher RSA for the phosphosite position when compared to the model in cyan.

Although phosphosites tend to have high all-atom relative surface accessibility (RSA) around 20% of the sites appear to be poorly accessible—here defined as having below 20% RSA. Given that surface accessibility should be a requirement for kinase regulation we explored potential explanations for these low accessibility sites. We hypothesized that this observation could be due to three potential factors: incorrect homology models, false positive phosphosites, or changes in protein conformation. We reasoned that if model quality was a determinant factor in explaining the inaccessible sites then the fraction of such sites should decrease with increasing quality of the models. However, homology models obtained from templates of higher sequence identity had a similar distribution of phosphosite RSA (Fig 2A). In order to test the impact of false positive sites we relied on the idea that conserved phosphosites are very unlikely to be experimental false positives. We noted that conserved sites have a similar fraction of poorly accessible phosphosites than non-conserved sites and no clear trend of accessibility relative to conservation is apparent. Overall these results suggest that false positive phosphosites are unlikely to be a major determinant for low surface accessibility sites.

If inaccessible phosphosites are not mostly due to incorrect homology models or false positive sites then conformation flexibility might explain why some sites appear to have low accessibility. Phosphosites are well known to regulate protein function by controlling protein conformation [36–39]. Xin and colleagues have compared structures of the same proteins in their modified and un-modified form and noted that PTMs tend to be associated with changes in protein conformation [37]. In addition, a structural analysis of 7 protein structures using normal mode analysis suggested that low accessibility sites could, in some cases, become accessible by conformational changes [40]. These studies suggest that the phosphosites that appear to be poorly accessible may occur in regions of proteins that can become more accessible in a conformation that is not captured in the structural template used to create these models. In order to study this we analyzed phosphoproteins for which we had more than one model structure obtained from different templates. For each pair of comparative models we correlated the accessibility for all serine, threonine and tyrosine residues. Overall, there is a high correlation of all-atom RSA value for different templates (Fig 2B, r = 0.67). However, phosphorylated residues showed a significantly higher change in accessibility in different models when compared to non-modified residues (Fig 2C, p-value = 6x10^-6^, two sample Kolmogorov-Smirnov test). The median absolute change in accessibility is 7.1 for the phospho-acceptor residues, 11.55 for phosphosites and 12.3 for conserved phosphosites. This result suggests that phosphosites are more likely to be in regions that show high conformation flexibility across different structural models. Phosphorylation sites are known to preferentially occur in disordered regions [41,42]. It is possible that the high conformational variability of phosphosites could be due to higher flexibility and/or lower modeling quality of disordered loop. We used DISOPRED to predict protein disorder across all phosphosites. As expected, we observed that phosphosites predicted to be in disordered regions had a higher median change in accessibility when compared to ordered sites (14.6 versus 10.7, Fig 2C). However, phosphosites predicted to be within ordered regions still have a significantly higher change in accessibility when compared to all acceptor residues (Fig 2C, 10.7 versus 7.1, p-value 6x10^-4^, two-sample Kolmogorov-Smirnov test). Phosphorylation can, in some cases, regulate protein conformation [43] and this analysis suggests that it is possible to use comparative models to define a class of functional phosphosites that can play a role in conformation regulation.

We analyzed in more detail sites that were at positions with large changes in accessibility across different templates and where the site had RSA below 20% in at least one model. Three of such cases are shown in Fig 2D were we superimposed the two models highlighting the differences in accessibility. YBX1 and YBX2 are RNA binding proteins and are phosphorylated in the cold-shock protein (CDP) domain (PFAM:PF00313) in a loop region (Fig 2D, left) that is known to be highly flexible and does not appear to play a direct role in RNA binding [44]. We cannot discount the possibility that large changes in accessibility in such large flexible loops are due to difficulties in modeling such protein regions. The Ran GTPase was also observed to be phosphorylated in a position with different accessibility in different templates (Fig 2D, center). The phosphosite is in a position equivalent to S135 in human RAN that has been previously shown to be regulated by p21-activated kinase 4 (PAK4) during the cell-cycle in human and *X*. *laevis* [45]. In addition, Ran S135 alanine and phosphomimetic mutants had an impact on microtubule nucleation in *X*. *laevis* extracts and in binding the exchange factor RCC1 in human cell lines [45]. It is possible that PAK4 regulation of S135 could promote a conformation that does not favor the interaction with RCC1. The third example is a phosphosite on the Dynamin 1-Like pleckstrin homology (PH) domain that comprises the “foot” region of dynamin like proteins (Fig 2D, right). The PH domain can adopt different conformations relative to the rest of the protein and the phosphorylated position changes drastically in accessibility depending on the conformation. The phosphorylated position is equivalent to the S635 in rat Drp1 and S616 in human Drp1 that has been previously shown to be regulated during cell-cycle and have functional roles in mitochondrial fission and microtubule targeting [46,47]. We hypothesize that the phosphosite may regulate Drp1 function by restricting the possible conformation variability of the PH domain relative to the rest of the protein. Although these examples would require experimental validation they illustrate how the structural analysis of phosphosites using different structural templates might suggest mechanist explanations for the function of MS identified sites.

### Degree of conservation in predicted kinase-protein interactions correlates with known and functionally meaningful phospho-regulatory events

**Fig 3:**
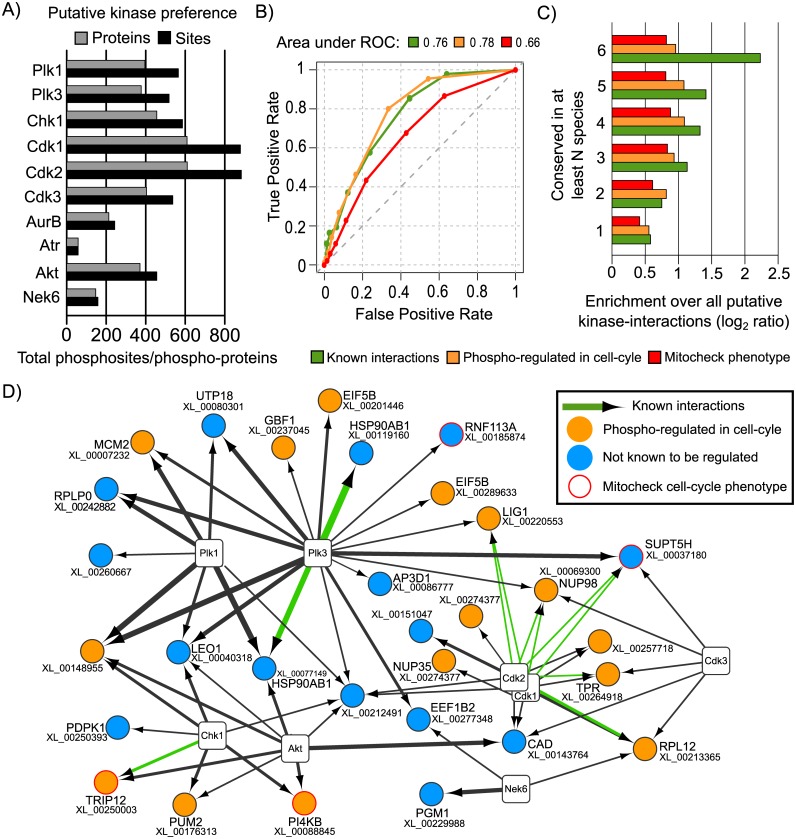
Conservation of putative cell-cycle related kinase interactions is predictive of known and/or cell-cycle related kinase-target interactions. A) The number of predicted kinase target sites and proteins associated with cell-cycle kinases selected for analysis in *X*. *laevis*. We tested if the degree of conservation of kinase-interactions was predictive of known interactions; enriched in proteins that are phospho-regulated in the cell cycle; and genes known to cause cell cycle phenotypes when knocked down. B) ROC curves measuring the accuracy for kinase-interaction predictions. C) Enrichment over random prediction for the 3 tested features. D) Predicted kinase interactions conserved in 7 or more species are shown, highlighting known interactions, proteins phospho-regulated during the cell cycle and genes causing cell cycle phonotypes. The edge thickness is proportional to the degree of conseravtion for the predicted kinase-protein interactions. A list of these interactions is provided in S4 Table.

Previous studies have shown that the conservation of kinase sequence motifs in alignments of orthologous proteins could be used to improve the predictions of kinase target sites [18,19]. However, some kinases are known to regulate proteins in clusters of sites [48–50] and in some cases it is plausible that the exact position of the target site might change within the protein during evolution yet maintaining the kinase-protein regulatory interaction [7,12,51,52]. In line with this reasoning, predicted kinase-protein interactions were often found to be conserved across species even when the phosphosite positions were not conserved [7]. We hypothesize that the conservation of predicted kinase-protein interactions across the large number of species analyzed here could be used to predict functionally important interactions. We decided to test this hypothesis focusing on cell-cycle kinases which allowed us to take advantage of previous large scale studies of cell cycle phosphoregulation [53] and phenotypes [54] for benchmarking purposes. To predict the targets of cell cycle-related kinases, we selected 16 kinases with many known target sites (Akt, Atr, AurA, AurB, Cdk1, Cdk2, Cdk3, Cdk7, Chk1, Nek2, Nek6, Nek9, Plk1, Plk2, Plk3, Ttk). Position specific scoring matrices (PSSMs) were derived for each kinase and benchmarked on a set of known target sites using cross validation (Methods and S2 Fig). From the 16 kinases we then selected 10 that had a cross validation area under the ROC curve (AROC) >0.7 (Akt, Atr, AurB, Cdk1, Cdk2, Cdk3, Chk1, Nek6, Plk1, Plk3, S3 Fig). For each species we predicted which phosphosites match the kinase preference of the 10 kinases studied. A phosphoprotein was predicted to be regulated by a kinase if at least one phosphosite was a predicted target of that kinase. The number of *X*. *laevis* sites and proteins predicted to be regulated by each kinase is shown in Fig 3A.

We next tested if the degree of conservation of kinase interactions is a useful predictor for known and functionally important kinase-target interactions. A putative *X*. *laevis* kinase-protein interaction was considered to be conserved in another species if an ortholog in that species had at least one phosphosite that was predicted to be a target of the same kinase model. We note that the sites do not have to be in the same region of the orthologous proteins. We ranked *X*. *laevis* kinase interactions according to the degree of conservation in the other 13 species. We then measured the enrichment of known or functionally important kinase-interactions relative to all predicted *X*. *laevis* kinase-protein interactions as a function of the degree of conservation. We observed that the degree of conservation was a significant predictor of previously described human kinase-protein interactions (Fig 3B AROC = 0.76 and 3C, “known interactions”). Highly conserved kinase-protein interactions were also enriched in target proteins that were previously known to be phosphoregulated during the cell cycle in human [53] (Fig 3B AROC = 0.78 and 3C, phospho-regulated) and enriched in genes that when knocked-down cause cell cycle-related phenotypes [54] (Fig 3B AROC = 0.66 and 3C, mitocheck phenotype). To exclude the possibility that the enrichment is due to the high scoring sequences that may be orthologous to the known human target sites of these kinases we repeated the analysis excluding all phosphosites that are 100% identical to known target sites of each kinase. No significant differences in the enrichment were observed as measured by the AROC curves (S4 Fig). To facilitate the re-use of these predictions we annotated 68 putative kinase-interactions conserved in 7 or more species (Fig 3D, S4 Table). When compared to all putative kinase-interactions this network is 6.8-fold enriched in known kinase-interactions (Fig 3C and 3D green arrows), 1.9-fold enriched in cell cycle phospho-regulated proteins (Fig 3C and 3D orange circles) and 1.3-fold enriched in genes associated with cell cycle-related phenotypes (Fig 3C and 3D red outline).

## Discussion

To facilitate the study of *X*. *laevis* PTM signaling, we obtained here a current survey of this species’ phosphoproteome. Previous studies have indicated that phosphoregulation can diverge quickly during evolution and that a fraction of phosphosites might serve no biological function in extant species. We used a compilation of phosphorylation data for 13 other species to identify highly conserved phosphosites and potential kinase-protein interactions. While others have shown that conservation of kinase sequence motifs across orthologous positions is a useful filter to predict kinase interactions [18,19] we show here that the degree of conservation of experimentally determined phosphorylation states is a strong predictor of sites with known function, of kinase-protein interactions and of function specific annotations (i.e. cell-cycle regulated and phenotypes). A small fraction of sites appear to be conserved over a large number of species. Given the divergence times separating the species studied here, these phosphosites likely have very ancient origins despite the fast evolutionary turn-over of phospho-regulation. As the experimental data on protein phosphorylation is incomplete for most species the conservation values presented here are under-estimated. Additional data will allow for further identification of such “ultra-conserved” and functionally important sites. Given that the collection of phosphorylation data across most species does not take into account different environmental or developmental conditions, these highly conserved sites are potentially biased for phosphosites that are constitutively on. Also, due to MS bias for higher protein abundance, highly conserved phosphosites described here are also potentially biased for proteins of higher abundance.

Although conservation is useful predictor of functional phosphosites, there are functionally important sites that are not highly conserved. For these reasons it is important to develop approaches that do not rely on conservation to rank PTMs according to biological importance. In this context, we and others have previously made use of structural information to predict PTMs that have the potential to regulate protein-protein interactions [13,20]. Using comparative models for *X*. *laevis* phosphoproteins we observed that some phosphosites appear to be in inaccessible regions and that phosphosites tend to be in positions of higher variability in surface accessibility across different structural templates. Our analysis suggests that structural models can therefore be used to predict, in an unbiased way, PTMs with the potential to regulate protein conformation. It will be important to verify this finding across different species and other PTM types. This approach could be further extended by including other structure based approaches such as normal mode analysis [40] and molecular dynamics [55] as well as sequence based approaches such as statistical coupling analysis [56].

The majority of PTMs identified to date for human and other species has no known function. Given the large throughput of MS approaches and the low fraction of PTMs with currently known functions much additional effort needs to be committed to the development of computational and experimental methods to elucidate PTM function. The evolutionary and structural observations presented here can be used to facilitate the prioritization of PTM functional studies in any species.

## Materials and Methods

### Ethics statement

All animal work was conducted according to relevant national and international guidelines. Animal protocols were approved by the Stanford University Administrative Panel on Laboratory Animal Care.

### Xenopus extract preparation

Frog eggs were obtained from female Xenopus laevis as described in [57,58]. Briefly, frogs were primed by injecting 50U of pregnant mare serum gonadotropin [PMSG) into the dorsal sacs 72h before egg collection. Egg laying was induced by injecting 500U of human chorionic gonadotropin (HCG) 18h before egg collection. 50ml of laid eggs were used for preparing interphase and mitotic extracts. A total of 500ul (at 20mg/ml) of interphase egg extracts supplemented with an ATP regenerating system and cycloheximide (100mg/ml) was prepared in the presence of protease inhibitors (leupeptin, pepstatin, cytochalasin and chymostatin). Half of the interphase extract was quick freeze for MS analysis. In order to make mitotic egg extracts, 250ul of interphase egg extracts were treated with 100nM non-degradable Xenopus D65-cyclin B1 and reactions were incubated for 1 hour and 30min at 22C. De-membranated sperm chromatin was added at 500/μl and samples were collected and stained with DAPI in order to monitor nuclear morphology and mitotic progression by fluorescence and phase microscopy. The criteria for M phase entry were condensed chromatin and a lack of a discernable nuclear envelope in at least 90% of the nuclei.

### Sample preparation for mass spectrometry analysis

Xenopus extracts were denatured in a buffer containing 8M urea, 0.1M Tris pH 8.0, and 150 mM NaCl. Disulfide bonds were reduced by incubation with 4 mM TCEP for 30 minutes at room temperature, and free sulfhydryl groups were alkylated by incubation with 10 mM iodoacetamide for 30 minutes at room temperature in the dark. Samples were diluted back to 2 M urea by addition of 0.1 M Tris pH 8.0, and trypsin was added at an enzyme:substrate ratios of 1:100. Lysates were digested overnight at 37 degrees Celsius. Following digestion the samples were concentrated using SepPak C18 cartridges (Waters). The C18 cartridge was washed once with 1 mL of 80% ACN, 0.1% TFA, followed by a 3 mL wash with 0.1% TFA. 10% TFA was added to each samples to a final concentration of 0.1% after which the samples were applied to the cartridge. The cartridge was washed with 3 mL of 0.1% TFA after binding, and the peptides were eluted with 40% ACN, 0.1% TFA. Following elution the peptides were lyophilized to dryness. Phosphopeptides were fractionated using hydrophilic interaction chromatography (HILIC) adapted from a method published by McNulty and Annan [59]. Buffers used for HILIC separation were HILIC buffer A (2% ACN, 0.1% TFA) and HILIC buffer B (98% ACN, 0.1% TFA). Peptides were resuspended in 90% HILIC buffer B and loaded onto a TSKgel amide-80 column (Tosoh Biosciences, 4.6 mm I.D. x 25 cm packed with 5 um particles). Peptides were separated at a flow rate of 0.5 mL / min using a gradient from 90% to 85% HILIC buffer B for 5 minutes, 85% to 55% HILIC buffer B for 80 minutes, then 55% to 0% HILIC buffer B for 5 minutes. Fractions were collected every 2 minutes and the 22 fractions previously determined to contain the majority of phosphopeptides were evaporated to dryness. Following HILIC fractionation, fractions were further enriched for phosphopeptides using titanium dioxide magnetic beads (Pierce) using the manufacturer’s protocol. Following titanium dioxide enrichment, sample were evaporated to dryness and resuspended in 0.1% formic acid for mass spectrometry analysis.

### Mass spectrometry analysis and phosphosite identification

Each fraction was analyzed separately by a Thermo Scientific LTQ Orbitrap Elite mass spectrometry system equipped with an Easy-nLC 1000 HPLC and autosampler. Samples were injected directly onto a reverse phase column (25 cm x 75 um I.D. packed with ReproSil-Pur C18-AQ 1.9 um particles) in buffer A [0.1% formic acid) at a maximum pressure of 800 bar. Peptides were separated with a gradient from 0% to 5% buffer B (100% ACN, 0.1% formic acid) over 5 minutes, then 5% to 30% buffer B over 52 minutes, then 30% to 95% buffer B over 1 minute, then held at 95% buffer B for 6 minutes. The separation was performed at a flow rate of 400 nl/min. The mass spectrometer continuously collected spectra in a data-dependent manner, acquiring a full scan in the Orbitrap (at 120,000 resolution with an automatic gain control target of 1,000,000 and a maximum injection time of 100 ms) followed by collision-induced dissociation spectra for the 20 most abundant ions in the ion trap (with an automatic gain control target of 10,000, a maximum injection time of 10 ms, a normalized collision energy of 35.0, activation Q of 0.250, isolation width of 2.0 m/z, and an activation time of 10.0). Singly and unassigned charge states were rejected for data-dependent selection. Dynamic exclusion was enabled to data-dependent selection of ions with a repeat count of 1, a repeat duration of 20.0 s, an exclusion duration of 20.0 s, an exclusion list size of 500, and exclusion mass width of + or—10.00 ppm. Raw mass spectrometry data was converted to peaklists using the PAVA algorithm. Data were searched using the Protein Prospector suite of algorithms (prospector.ucsf.edu). The data were searched against a *X*. *laevis* proteome obtained from the genome sequencing project. Specifically, we used a version containing 24,762 gene models obtained from sequencing of tissue samples and containing the longest gene model for each putative orthologous group. ("OrthoGeneOne" model of Taira201203_XENLA_tissue data, available at http://www.marcottelab.org/index.php/XENLA_GeneModel2012). Searches were run with a concatenated decoy database comprised of all sequences with their amino acids randomized. The algorithm searched for fully tryptic peptides with up to 2 missed cleavages using a parent mass tolerance of 20 ppm and a fragment mass tolerance of 0.8 Da. The algorithm indicated a static modification for carboxyamidomethyl of cysteine residues, and for variable modifications acetylation of protein N-termini, glutamine to pyroglutamate conversion, methionine oxidation, and phosphorylation of serine, threonine, or tyrosine residues. Data were filtered using a Protein Prospector expectation value that was resulted in a false discovery rate of 1% as determined by the number of matches to the randomized decoy database. The number of phosphosites identified in each HILIC fraction are provided in S5 Table. The phosphosite localization within the peptide was scored using the SLIP score [30]. For most of the analysis we made use of all phosphosites, included those that are ambiguously localized within the peptide sequence. A higher confidence list of well localized sites was generated by selected phosphosites with an Evalue<0.001 and a SLIP score > = 3. Benchmarks for the SLIP score suggest that at this cut-off over 90% of the sites are well localized. The list of identified phosphopetides and corresponding quality scores is provided in S1 Table.

### Data analysis

Putative orthologs of *X*. *laevis* phosphoproteins were predicted using the reciprocal best-BLAST hits method [60] against a set of 13 proteomes with currently available phosphorylation information. Putative orthologs were aligned using MUSCLE [61]. The phosphorylation information for the 13 species was retrieved from the ptmfunc database (http://ptmfunc.com) and includes phosphosite information for *Saccharomyces cerevisiae*, *Schizosaccharomyces pombe*, *Plasmodium falciparum*, *Toxoplasma gondii*, *Trypanosoma brucei*, *Trypanosoma cruzi*, *Oryza sativa*, A*rabidopsis thaliana*, *Drosophila melanogaster*, *Caenorhabditis elegans*, *Homo sapiens*, *Rattus norvegicus* and *Mus musculus*. An *X*. *laevis* phosphosite was considered to be conserved in a target species if the predicted orthologous protein was known to be phosphorylated in the target species in a window of +/-2 residues around the aligned position. A window was used to take into account the alignment uncertainty and the ambiguity in identifying the exact position of the phosphorylated residue within a phosphopeptide. To predict the targets of cell cycle-related kinases, we selected 16 kinases (Akt, Atr, AurA, AurB, Cdk1, Cdk2, Cdk3, Cdk7, Chk1, Nek2, Nek6, Nek9, Plk1, Plk2, Plk3, Ttk) to train specificity models based on known target site data. Kinase substrate data for 357 kinases was obtained from public databases (PhosphoSitePlus [62], PhosphoELM [63] and HPRD [64]). In total we collected 9,595 kinase substrate relationships (KSR), based on 6,747 kinase-associated phosphosites. The positive set of phosphosites for a given kinase model was defined as the set of phosphosites annotated to that kinase, whereas the negative set is defined as sites annotated to any other kinase. Position specific scoring matrices (PSSMs) were derived for each of the 16 kinases and benchmarked on a set of known target sites using a cross-fold validation and area under the ROC curve (S2 Fig). From the 16 kinases we then selected 10 that had a cross-fold validation AUC>0.7 (Akt, Atr, AurB, Cdk1, Cdk2, Cdk3, Chk1, Nek6, Plk1, Plk3). Each model was used to score the corresponding positive and negative sets of peptides using the Matrix Similarity Score (MSS) as described in [65] and the MSS threshold that maximised the accuracy was used. Positive and negative sample sizes are required to be similar if not equal; therefore, the positive set was up sampled with replacement to the size of the negative sample, prior to computing the accuracy. MSS cutoffs for each kinase is as follows: Plk1 (0.436), Plk3 (0.386), Chk1 (0.236), Cdk1 (0.929), Cdk2 (0.126), Cdk3 (0.455), AurB (0.14), Atr (0.932), Akt (0.662), Nek6 (0.402). Structural models of *X*. *laevis* phosphoproteins were built automatically using ModPipe [31] relying on Modeller 9.10 [66]. A model was considered acceptable if the template sequence identify was at least 25% and met one additional criterion: TSVMod NO35 > = 40%, GA341 > = 0.7, E-value <0.0001 or zDOPE <0. All-atom residue relative surface accessibility was computed using NACCESS [67]. A list of models created with selected PDB codes and model quality values are available in S6 Table.

## Supporting Information

S1 Fig Enrichment of functional phosphorylation events as a function of conservation. For each *X*. *laevis* phosphosite we counted the number of species in which the orthologous peptide region is also phosphorylated. We excluded all phosphosites that are not also phosphorylated in human. We then calculated the fraction of sites that are known to play a functional role in human. The degree of conservation is found to enrich significantly for sites with a known function for all *X*. *laevis* sites as well as sites that are in ordered or disordered regions.
(PDF)

S2 Fig Benchmark for kinase specificity position specific scoring matrices. For 16 cell-cycle kinases with many known target sites (Akt, Atr, AurA, AurB, Cdk1, Cdk2, Cdk3, Cdk7, Chk1, Nek2, Nek6, Nek9, Plk1, Plk2, Plk3, Ttk), position specific scoring matrices (PSSMs) were derived for each kinase and benchmarked on a set of known target sites using a cross-fold validation 16. We show here the AROC curves for the cross validation for each kinase.
(PDF)

S3 Fig Median values for cross-validation AROC values for 16 cell-cycle kinases with many known target sites (Akt, Atr, AurA, AurB, Cdk1, Cdk2, Cdk3, Cdk7, Chk1, Nek2, Nek6, Nek9, Plk1, Plk2, Plk3, Ttk). Those with AROC > 0.7 were selected for further studies.
(PDF)

S4 Fig ROC curves measuring the accuracy for kinase-interaction predictions. We tested if the degree of conservation of kinase-interactions was predictive of known interactions; enriched in proteins that are phospho-regulated in the cell cycle; and genes known to cause cell cycle phenotypes when knocked down. For this analysis we removed any phosphopeptide in all species that was 100% identical to a human known target site of each tested kinase.
(PDF)

S1 Table List of experimentally identified phospho-peptides. Mass-spectrometry derived phosphopeptides are described in accompanying spreadsheet.
(XLS)

S2 Table Estimation of well-localized sites in the MS phosphorylation dataset. We obtained the largest SLIP score observed for each non-redundant phosphosite position and binned the set of phosphosites according the SLIP scores. Non ambiguous sites are those that are observed in phosphopeptides with just a single acceptor residue and are therefore well localized. Fully ambiguous are those that have acceptor residues within the phosphopeptide with probabilities that are not distinguishable. For all SLIP score bins we obtained the local false localization rate (FLR) from a benchmark study [30] and used this to estimate the number of sites that not well localized in each bin. We estimate that the dataset we collected has 76.7% of sites well localized.
(DOC)

S3 Table List of species used for comparative analysis. For each species we list the count of phosphorylation sites obtained from PTMfunc, the putative orthologs relative to *X*. *laevis* and total phosphosites within the list of putative orthologous proteins.
(DOC)

S4 Table List of predicted kinase interactions conserved in 7 or more species. Cell cycle regulated: human protein known to have phosphosites that are regulated during the cell-cycle (1 –yes; 0 –no). Mitocheck pheno:human gene known to cause a mitotic phenotype as described in the Mitocheck database (1 –yes; 0 –no); TP—Known human kinase-substrate interaction as described in the PhosphositePlus database (1 –yes; 0 –no). n_cons: Number of species with a conserved predicted kinase-protein interaction.
(DOC)

S5 Table Number of phosphopeptides identified per HILIC fraction. (DOC)

S6 Table List of comparative models created for *X*. *laevis* phosphoproteins. Structural models of *X*. *laevis* phosphoproteins were built automatically using ModPipe relying on Modeller 9.10. A model was considered acceptable if the template sequence identify was at least 25% and met one additional criterion: TSVMod NO35 > = 40%, GA341 > = 0.7, E-value <0.0001 or zDOPE <0.
(XLS)
