# Supplementary figures and images for: Prediction of Functionally Important Phospho-Regulatory Events in Xenopus laevis Oocytes
Source: PLoS Comput Biol. 2015 Aug 27;11(8):e1004362. doi: 10.1371/journal.pcbi.1004362 (PMC4552029; doi:10.1371/journal.pcbi.1004362)

All sites

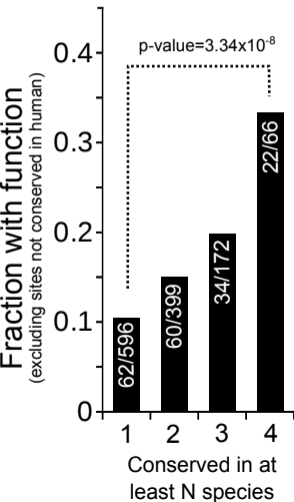

Disordered

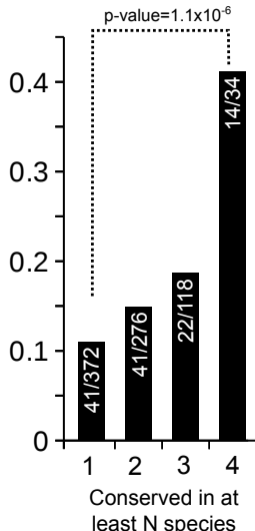

Ordered

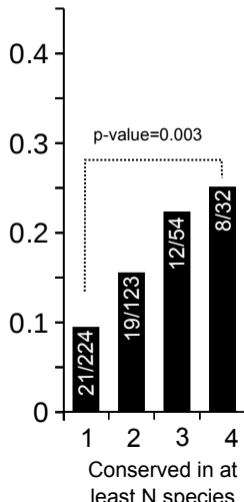

Supplement: S1 Fig — For each X. laevis phosphosite we counted the number of species in which the orthologous peptide region is also phosphorylated. We excluded all phosphosites that are not also phosphorylated in human. We then calculated the fraction of sites that are known to play a functional role in human. The degree of conservation is found to enrich significantly for sites with a known function for all X. laevis sites as well as sites that are in ordered or disordered regions. (PDF) [file pcbi.1004362.s001.pdf]

AUC

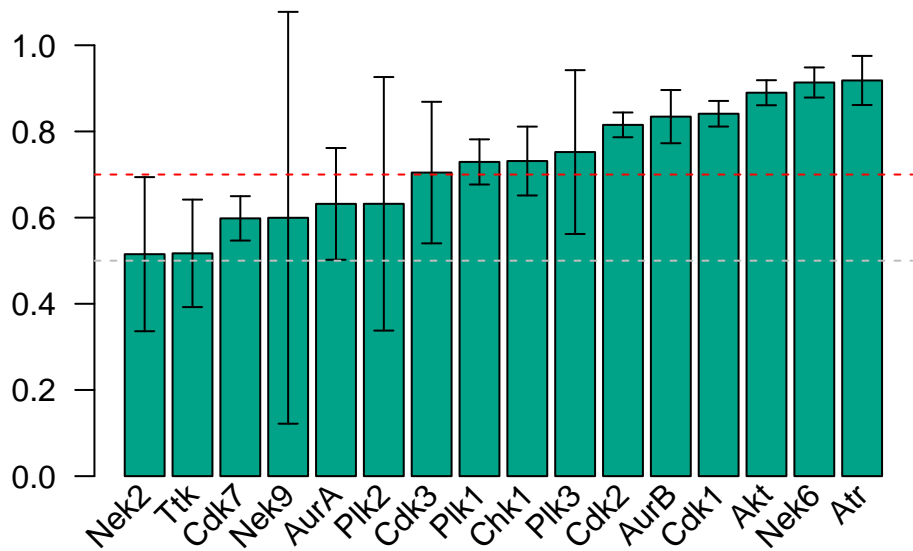

Supplement: S3 Fig — Those with AROC > 0.7 were selected for further studies. (PDF) [file pcbi.1004362.s003.pdf]

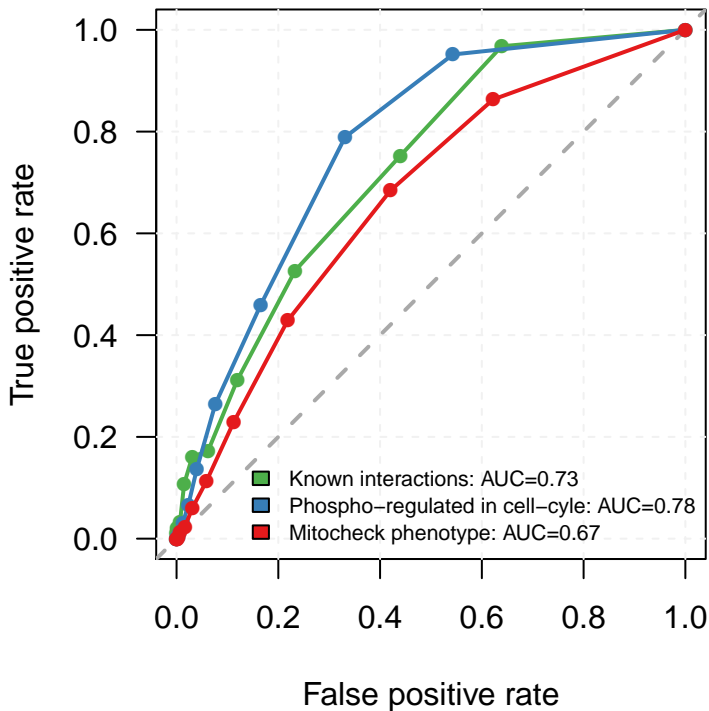

Supplement: S4 Fig — We tested if the degree of conservation of kinase-interactions was predictive of known interactions; enriched in proteins that are phospho-regulated in the cell cycle; and genes known to cause cell cycle phenotypes when knocked down. For this analysis we removed any phosphopeptide in all species that was 100% identical to a human known target site of each tested kinase. (PDF) [file pcbi.1004362.s004.pdf]
