# Supplementary material for: Prediction of Functionally Important Phospho-Regulatory Events in Xenopus laevis Oocytes
Source: PLoS Comput Biol. 2015 Aug 27;11(8):e1004362. doi: 10.1371/journal.pcbi.1004362 (PMC4552029; doi:10.1371/journal.pcbi.1004362)
Supplement: S2 Fig — For 16 cell-cycle kinases with many known target sites (Akt, Atr, AurA, AurB, Cdk1, Cdk2, Cdk3, Cdk7, Chk1, Nek2, Nek6, Nek9, Plk1, Plk2, Plk3, Ttk), position specific scoring matrices (PSSMs) were derived for each kinase and benchmarked on a set of known target sites using a cross-fold validation 16. We show here the AROC curves for the cross validation for each kinase. (PDF) [file pcbi.1004362.s002.pdf]

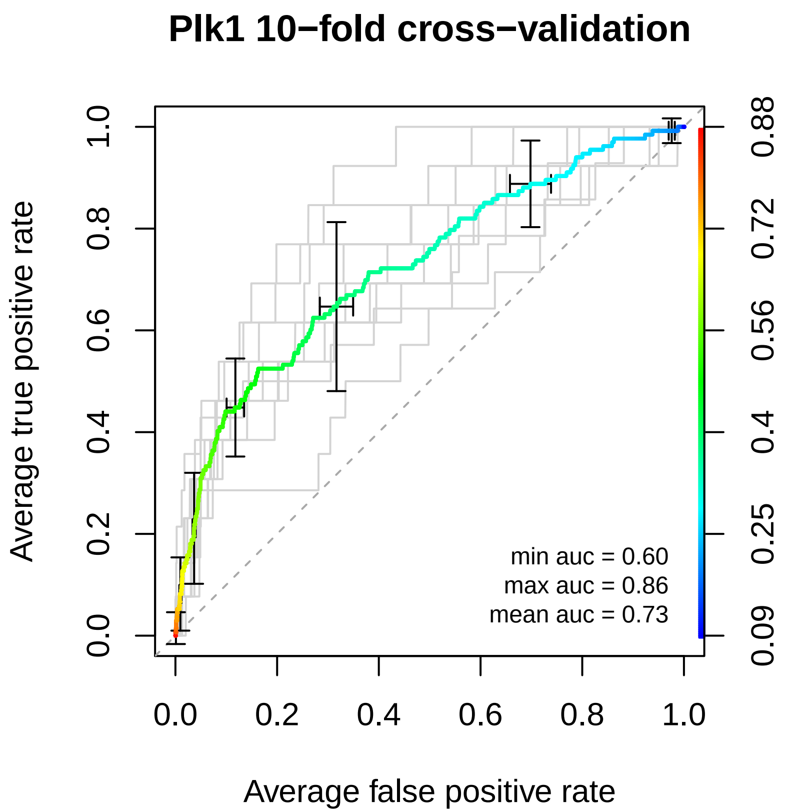
Plk1 10−fold cross−validation
Average true positive rate
Average false positive rate
0.0
0.2
0.4
0.6
0.8
1.0
0.09
0.25
0.4
0.56
0.72
0.88
min auc = 0.60
max auc = 0.86
mean auc = 0.73

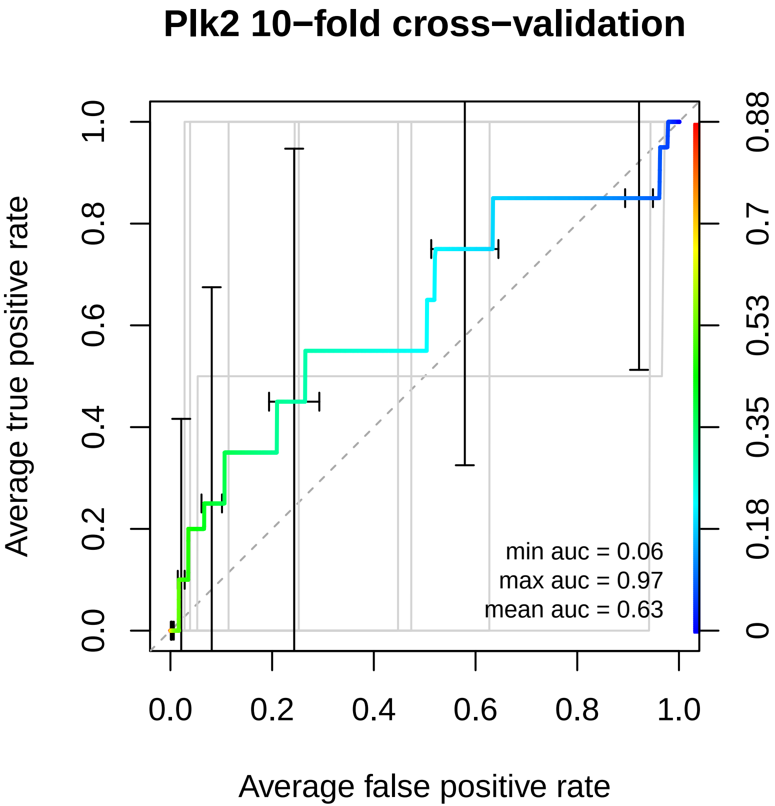
Plk2 10−fold cross−validation
Average true positive rate
Average false positive rate
0.0
0.2
0.4
0.6
0.8
1.0
0
0.18
0.35
0.53
0.7
0.88
min auc = 0.06
max auc = 0.97
mean auc = 0.63

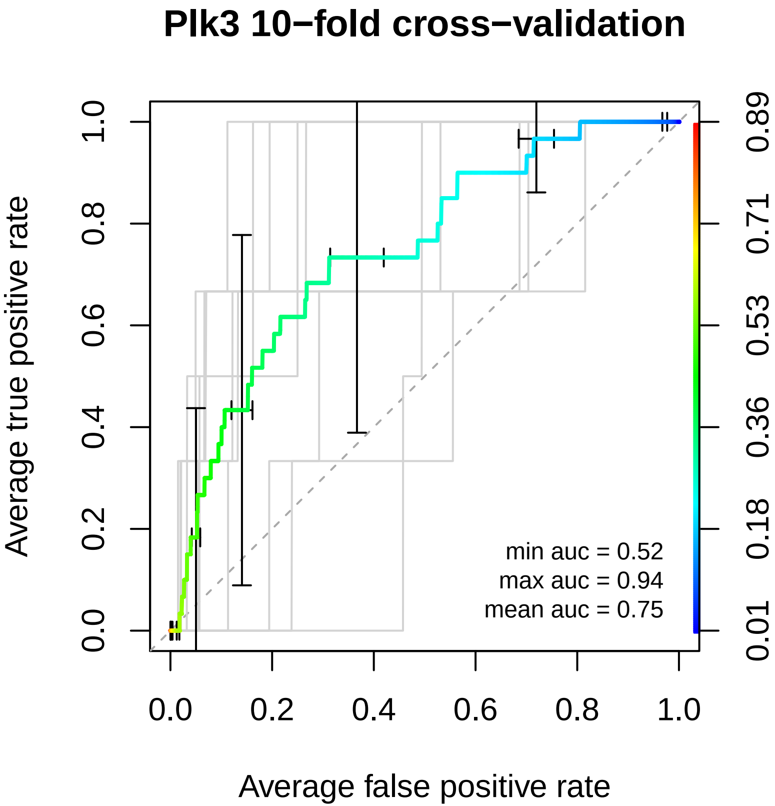
Plk3 10−fold cross−validation
Average true positive rate
Average false positive rate
0.0
0.2
0.4
0.6
0.8
1.0
0.01
0.18
0.36
0.53
0.71
0.89
min auc = 0.52
max auc = 0.94
mean auc = 0.75

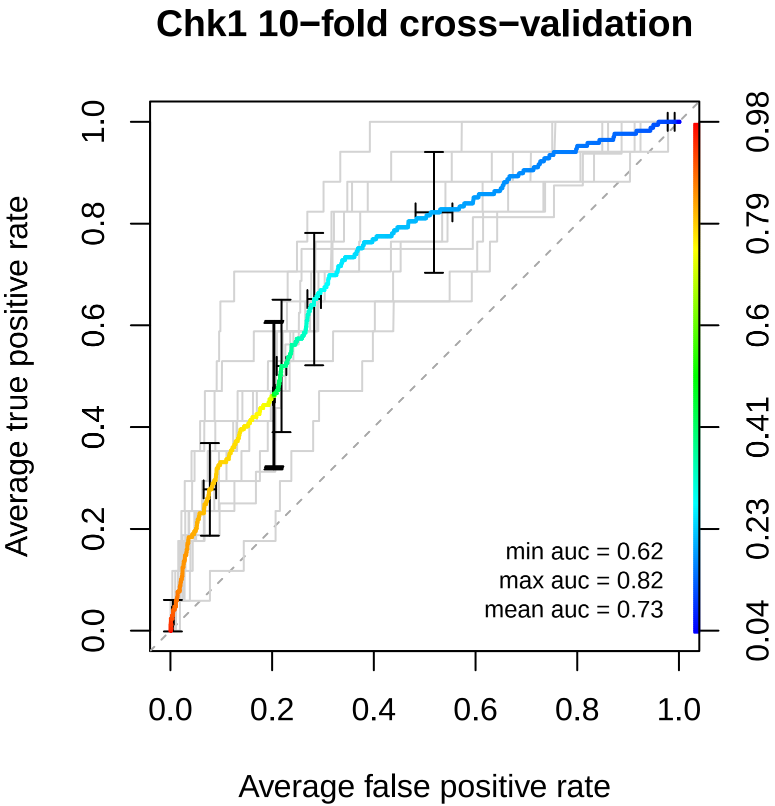
Chk1 10−fold cross−validation
Average true positive rate
Average false positive rate
0.0
0.2
0.4
0.6
0.8
1.0
0.98
0.79
0.6
0.41
0.23
0.04
min auc = 0.62
max auc = 0.82
mean auc = 0.73

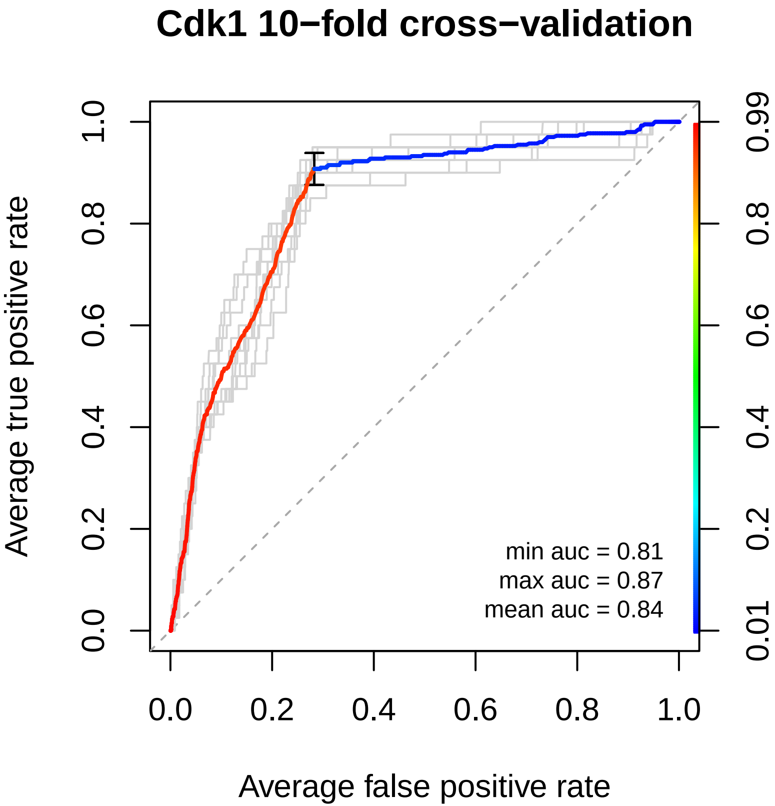

Cdk1 10−fold cross−validation
Average true positive rate
Average false positive rate
0.0
0.2
0.4
0.6
0.8
1.0
0.01
0.2
0.4
0.6
0.8
0.99
min auc = 0.81
max auc = 0.87
mean auc = 0.84

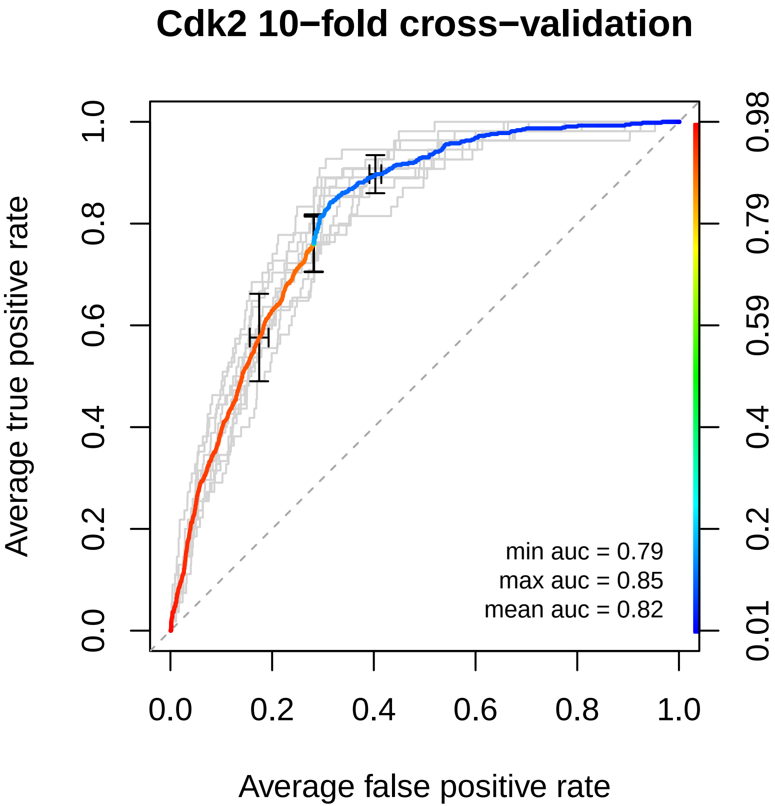

Cdk2 10−fold cross−validation
Average true positive rate
Average false positive rate
0.0
0.2
0.4
0.6
0.8
1.0
0.01
0.2
0.4
0.59
0.79
0.98
min auc = 0.79
max auc = 0.85
mean auc = 0.82

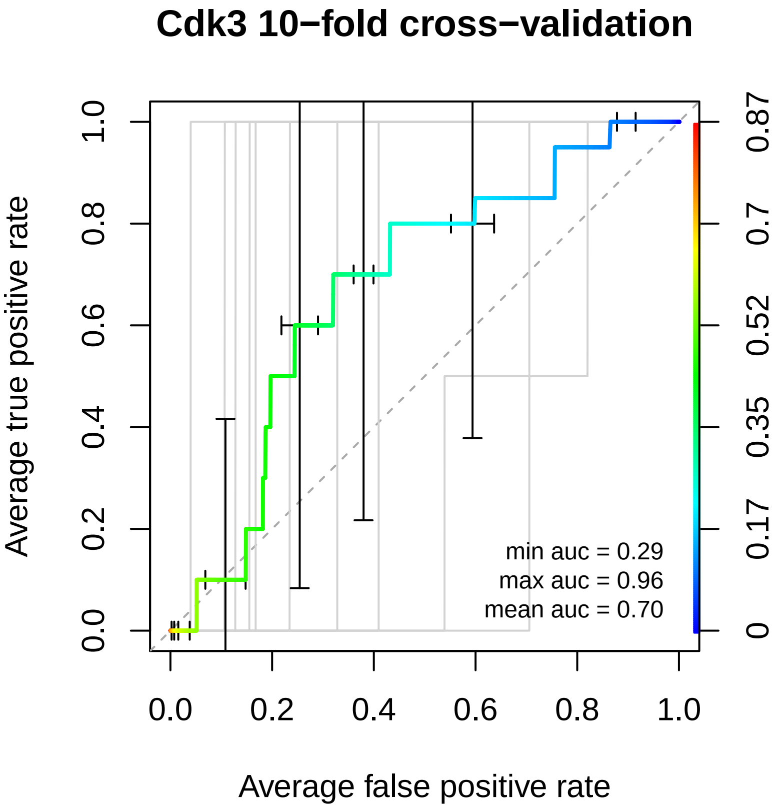

Cdk3 10−fold cross−validation
Average true positive rate
Average false positive rate
0.0
0.2
0.4
0.6
0.8
1.0
0
0.17
0.35
0.52
0.7
0.87
min auc = 0.29
max auc = 0.96
mean auc = 0.70

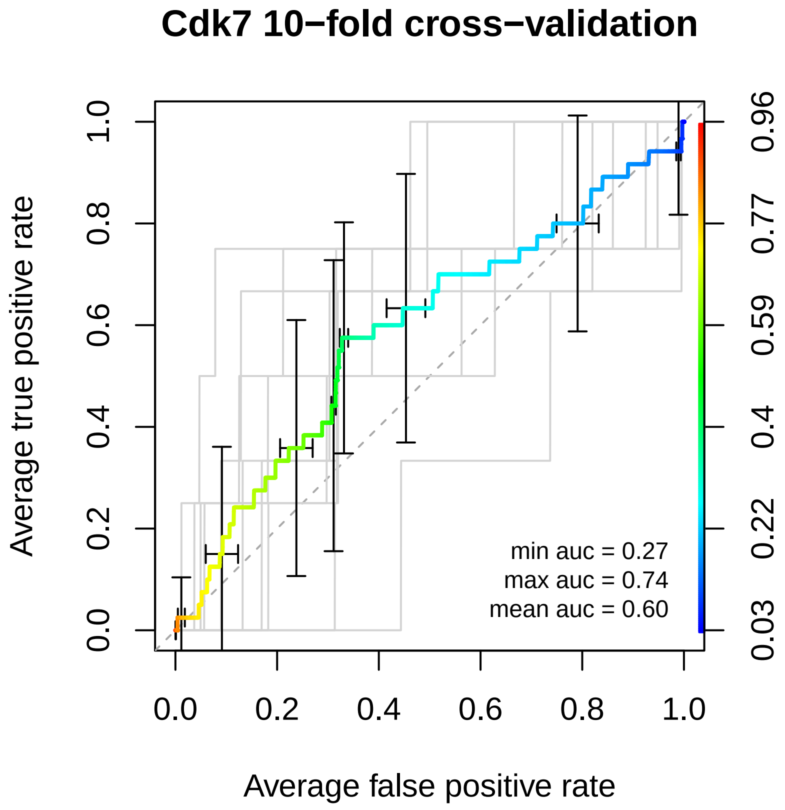
Cdk7 10−fold cross−validation
Average true positive rate
Average false positive rate
0.0
0.2
0.4
0.6
0.8
1.0
0.03
0.22
0.4
0.59
0.77
0.96
min auc = 0.27
max auc = 0.74
mean auc = 0.60

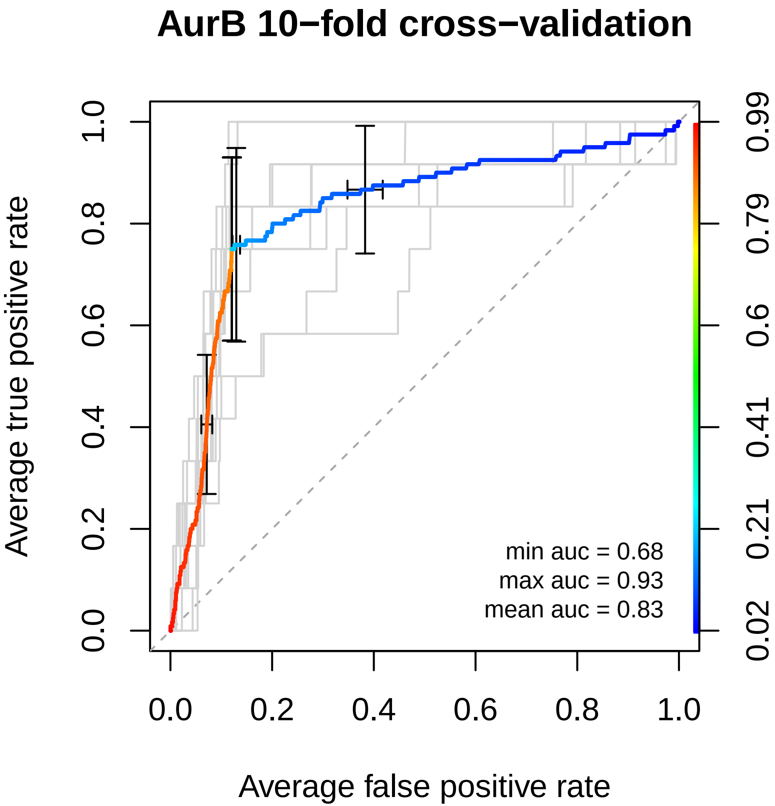

AurB 10−fold cross−validation
Average true positive rate
Average false positive rate
0.0
0.2
0.4
0.6
0.8
1.0
0.02
0.21
0.41
0.6
0.79
0.99
min auc = 0.68
max auc = 0.93
mean auc = 0.83

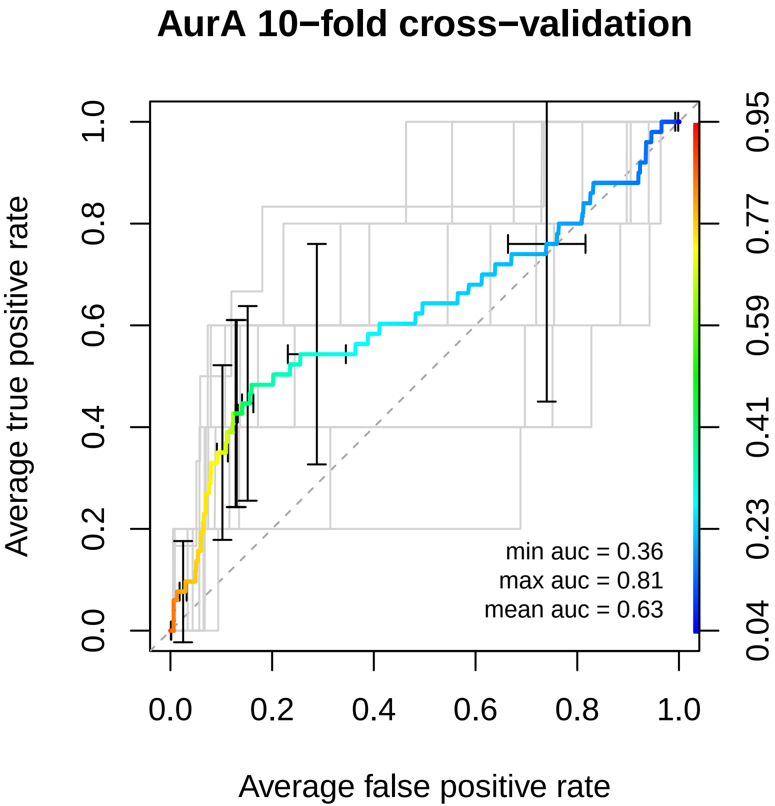
AurA 10−fold cross−validation
Average true positive rate
Average false positive rate
0.0
0.2
0.4
0.6
0.8
1.0
0.95
0.77
0.59
0.41
0.23
0.04
min auc = 0.36
max auc = 0.81
mean auc = 0.63

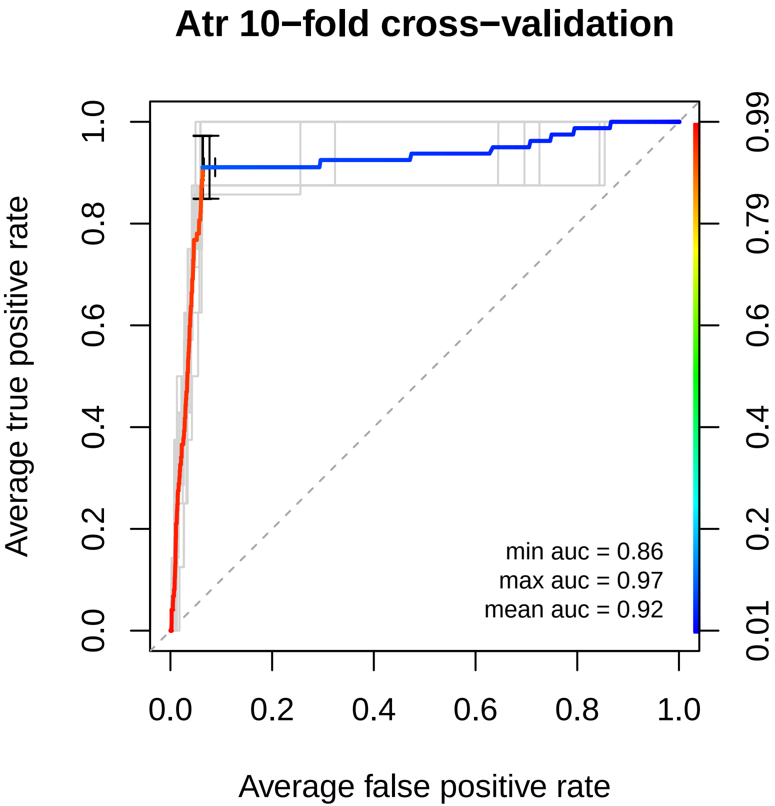
Atr 10−fold cross−validation
Average true positive rate
Average false positive rate
0.0
0.2
0.4
0.6
0.8
1.0
0.01
0.2
0.4
0.6
0.79
0.99
min auc = 0.86
max auc = 0.97
mean auc = 0.92

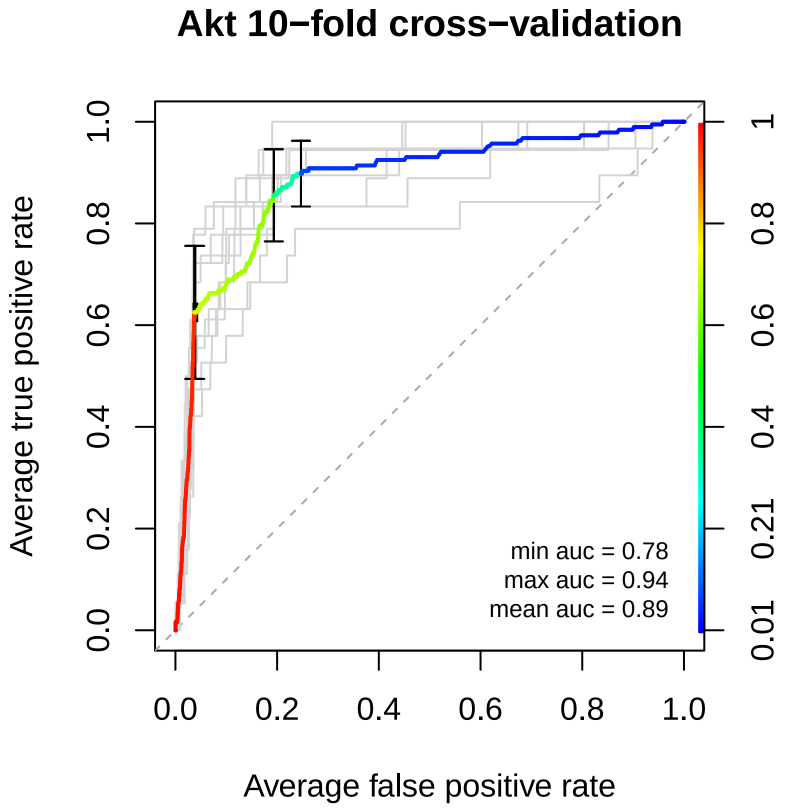

Akt 10−fold cross−validation
Average true positive rate
Average false positive rate
0.0
0.2
0.4
0.6
0.8
1.0
0.01
0.21
0.4
0.6
0.8
1
min auc = 0.78
max auc = 0.94
mean auc = 0.89

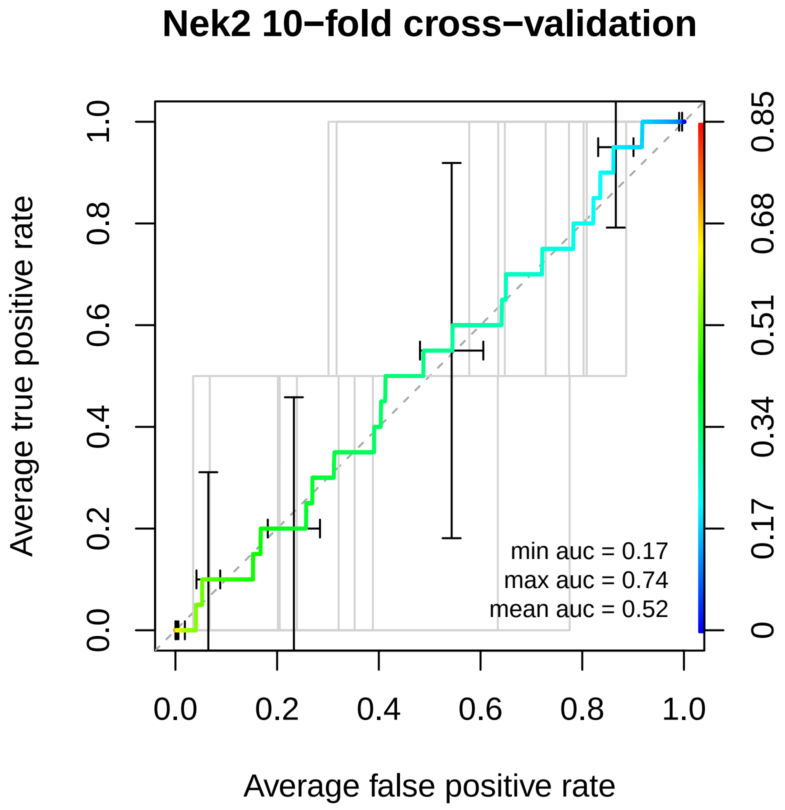

Nek2 10−fold cross−validation
Average true positive rate
Average false positive rate
0.0
0.2
0.4
0.6
0.8
1.0
0
0.17
0.34
0.51
0.68
0.85
min auc = 0.17
max auc = 0.74
mean auc = 0.52

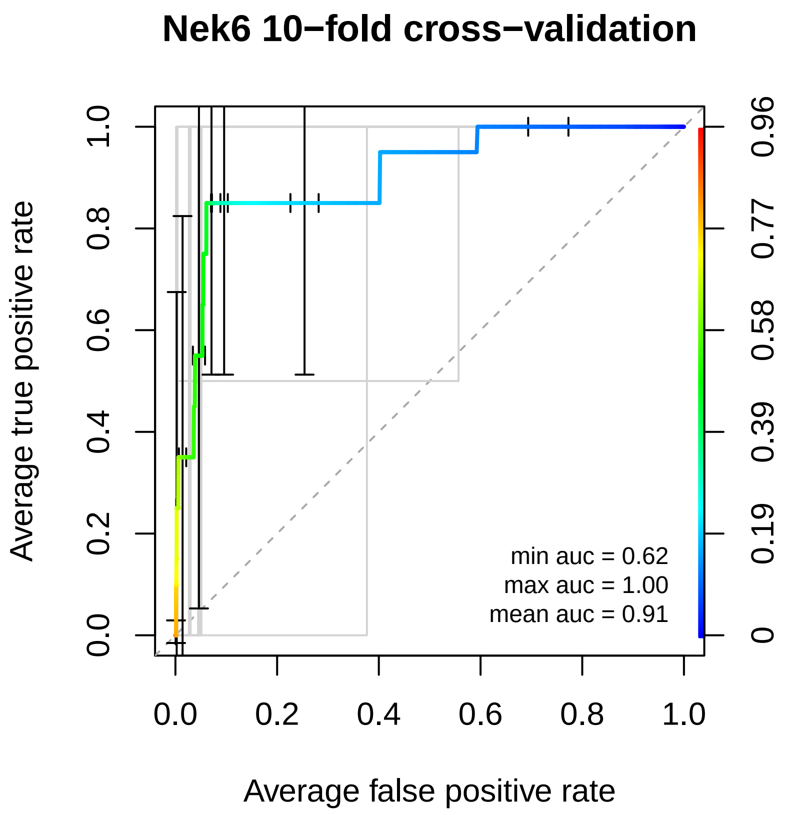
Nek6 10−fold cross−validation
Average true positive rate
Average false positive rate
0.0
0.2
0.4
0.6
0.8
1.0
0
0.19
0.39
0.58
0.77
0.96
min auc = 0.62
max auc = 1.00
mean auc = 0.91

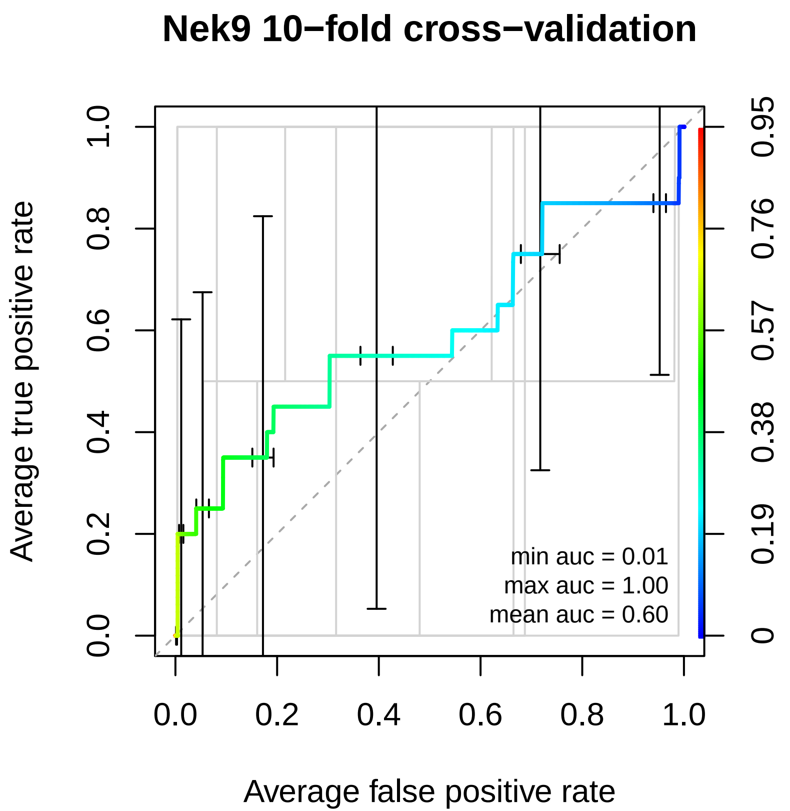
Nek9 10−fold cross−validation
Average true positive rate
Average false positive rate
0.0
0.2
0.4
0.6
0.8
1.0
0
0.19
0.38
0.57
0.76
0.95
min auc = 0.01
max auc = 1.00
mean auc = 0.60

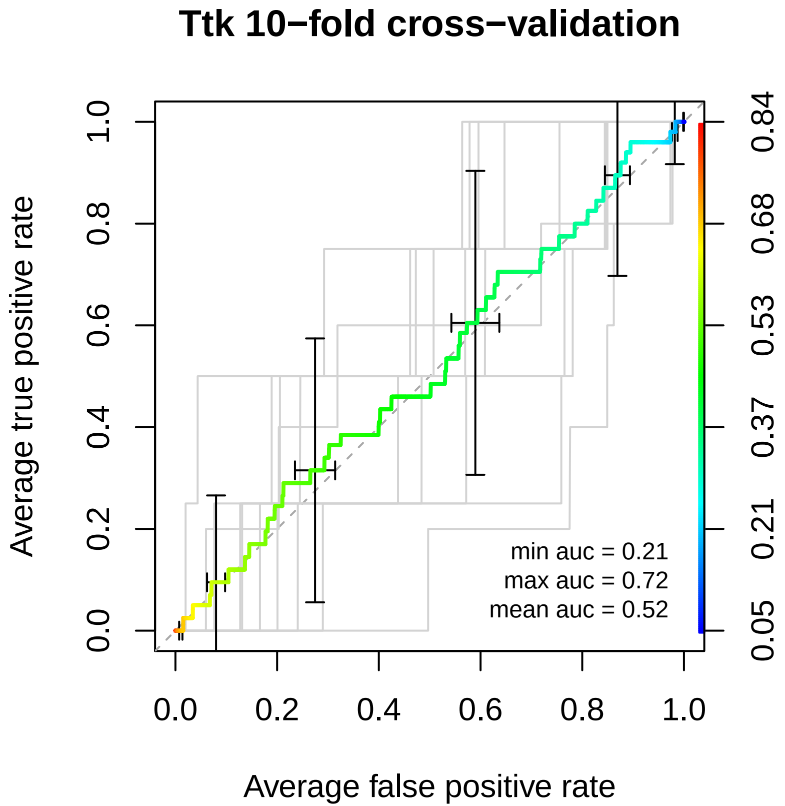

Ttk 10−fold cross−validation
Average true positive rate
Average false positive rate
0.0
0.2
0.4
0.6
0.8
1.0
0.05
0.21
0.37
0.53
0.68
0.84
min auc = 0.21
max auc = 0.72
mean auc = 0.52
