# Supplementary material for: Prediction of Functionally Important Phospho-Regulatory Events in Xenopus laevis Oocytes
Source: PLoS Comput Biol. 2015 Aug 27;11(8):e1004362. doi: 10.1371/journal.pcbi.1004362 (PMC4552029; doi:10.1371/journal.pcbi.1004362)
Supplement: S2 Table — We obtained the largest SLIP score observed for each non-redundant phosphosite position and binned the set of phosphosites according the SLIP scores. Non ambiguous sites are those that are observed in phosphopeptides with just a single acceptor residue and are therefore well localized. Fully ambiguous are those that have acceptor residues within the phosphopeptide with probabilities that are not distinguishable. For all SLIP score bins we obtained the local false localization rate (FLR) from a benchmark study [30] and used this to estimate the number of sites that not well localized in each bin. We estimate that the dataset we collected has 76.7% of sites well localized. (DOC) [file pcbi.1004362.s006.doc]

Supplementary Table 2 – Estimation of well-localized sites in the MS phosphorylation dataset. We obtained the largest SLIP score observed for each non-redundant phosphosite position and binned the set of phosphosites according the SLIP scores. Non ambiguous sites are those that are observed in phosphopeptides with just a single acceptor residue and are therefore well localized. Fully ambiguous are those that have acceptor residues within the phosphopeptide with probabilities that are not distinguishable. For all SLIP score bins we obtained the local false localization rate (FLR) from a benchmark study (30) and used this to estimate the number of sites that not well localized in each bin. We estimate that the dataset we collected has 76.7% of sites well localized.

| SLIP score | Count | FLR | Estimated false localizations |
| --- | --- | --- | --- |
| Non ambiguous | 159 | 0 | 0 |
| >=8 | 689 | 0 | 0 |
| 6 to 7 | 161 | 0.05 | 8.05 |
| 3 to 5 | 201 | 0.1 | 20.1 |
| 2 | 105 | 0.3 | 31.5 |
| 1 | 125 | 0.5 | 62.5 |
| 0 | 53 | 0.7 | 37.1 |
| Fully ambiguous | 245 | 1 | 245 |
| Total | 1738 |  | 404.25 |
