# Supplementary material for: Prediction of Functionally Important Phospho-Regulatory Events in Xenopus laevis Oocytes
Source: PLoS Comput Biol. 2015 Aug 27;11(8):e1004362. doi: 10.1371/journal.pcbi.1004362 (PMC4552029; doi:10.1371/journal.pcbi.1004362)
Supplement: S3 Table — For each species we list the count of phosphorylation sites obtained from PTMfunc, the putative orthologs relative to X. laevis and total phosphosites within the list of putative orthologous proteins. (DOC) [file pcbi.1004362.s007.doc]

Supplementary Table 3 – List of species used for comparative analysis. For each species we list the count of phosphorylation sites obtained from PTMfunc, the putative orthologs relative to *X. laevis* and total phosphosites within the list of putative orthologous proteins.

| Species | Total Phosphosites | Number of putative orthologs in species | Phosphosites in putative orthologs |
| --- | --- | --- | --- |
| *S. pombe* | 10057 | 2315 | 4460 |
| *D. melanogaster* | 22307 | 5378 | 9498 |
| *H. sapiens* | 70587 | 8990 | 43811 |
| *T. brucei* | 1738 | 1918 | 490 |
| *S. cerevisiae* | 20658 | 2104 | 7118 |
| *P. falciparum* | 4873 | 1521 | 1685 |
| *C. elegans* | 8433 | 4494 | 4325 |
| *A. thaliana* | 5336 | 3194 | 1130 |
| *O. sativa* | 3666 | 3216 | 1082 |
| *T. cruzi* | 2556 | 2095 | 253 |
| *T. gondi* | 13046 | 1926 | 3545 |
| *R. norvegicus* | 5268 | 10688 | 3812 |
| *M. musculus* | 32886 | 11248 | 24526 |
