# Supplementary material for: Prediction of Functionally Important Phospho-Regulatory Events in Xenopus laevis Oocytes
Source: PLoS Comput Biol. 2015 Aug 27;11(8):e1004362. doi: 10.1371/journal.pcbi.1004362 (PMC4552029; doi:10.1371/journal.pcbi.1004362)
Supplement: S4 Table — Cell cycle regulated: human protein known to have phosphosites that are regulated during the cell-cycle (1 –yes; 0 –no). Mitocheck pheno:human gene known to cause a mitotic phenotype as described in the Mitocheck database (1 –yes; 0 –no); TP—Known human kinase-substrate interaction as described in the PhosphositePlus database (1 –yes; 0 –no). n_cons: Number of species with a conserved predicted kinase-protein interaction. (DOC) [file pcbi.1004362.s008.doc]

Supplementary Table 4 – List of predicted kinase interactions conserved in 7 or more species. Cell cycle regulated: human protein known to have phosphosites that are regulated during the cycle (1 – yes; 0 – no). Mitocheck pheno:human gene known to cause a mitotic phenotype as described in the Mitocheck database (1 – yes; 0 – no); TP - Known human kinase-substrate interaction as described in the PhosphositePlus database (1 – yes; 0 – no). n_cons: Number of species with a conserved predicted kinase-protein interaction.

| Kinase | *X. laevis* protein | Putative human ortholog | Cell cycle regulated | Mitocheck pheno | TP | n_cons |
| --- | --- | --- | --- | --- | --- | --- |
| Plk3 | XL_00119160 | HSP90AB1 | 0 | 0 | 1 | 11 |
| Plk1 | XL_00077149 | HSP90AB1 | 0 | 0 | 0 | 10 |
| Plk3 | XL_00077149 | HSP90AB1 | 0 | 0 | 1 | 10 |
| Plk1 | XL_00148955 |  | 1 | 0 | 0 | 10 |
| Plk3 | XL_00148955 |  | 1 | 0 | 0 | 10 |
| Plk3 | XL_00080301 | UTP18 | 0 | 0 | 0 | 9 |
| Plk1 | XL_00007232 | MCM2 | 1 | 0 | 0 | 9 |
| Plk1 | XL_00242882 | RPLP0 | 0 | 0 | 0 | 9 |
| Plk3 | XL_00242882 | RPLP0 | 0 | 0 | 0 | 9 |
| Nek6 | XL_00229988 | PGM1 | 0 | 0 | 0 | 9 |
| Plk3 | XL_00040318 | LEO1 | 0 | 0 | 0 | 9 |
| Plk3 | XL_00037180 | SUPT5H | 0 | 1 | 0 | 9 |
| Akt | XL_00143764 | CAD | 0 | 0 | 0 | 9 |
| Plk1 | XL_00080301 | UTP18 | 0 | 0 | 0 | 8 |
| Plk3 | XL_00007232 | MCM2 | 1 | 0 | 0 | 8 |
| Chk1 | XL_00088845 | PI4KB | 1 | 1 | 0 | 8 |
| Akt | XL_00088845 | PI4KB | 1 | 1 | 0 | 8 |
| Akt | XL_00077149 | HSP90AB1 | 0 | 0 | 0 | 8 |
| Plk3 | XL_00201446 | EIF5B | 1 | 0 | 0 | 8 |
| Chk1 | XL_00148955 |  | 1 | 0 | 0 | 8 |
| Akt | XL_00148955 |  | 1 | 0 | 0 | 8 |
| Plk1 | XL_00040318 | LEO1 | 0 | 0 | 0 | 8 |
| Chk1 | XL_00040318 | LEO1 | 0 | 0 | 0 | 8 |
| Chk1 | XL_00176313 | PUM2 | 1 | 0 | 0 | 8 |
| Cdk2 | XL_00151047 |  | 0 | 0 | 0 | 8 |
| Chk1 | XL_00250003 | TRIP12 | 1 | 1 | 1 | 8 |
| Akt | XL_00250003 | TRIP12 | 1 | 1 | 0 | 8 |
| Plk3 | XL_00277348 | EEF1B2 | 0 | 0 | 0 | 8 |
| Cdk1 | XL_00213365 | RPL12 | 1 | 0 | 0 | 8 |
| Cdk2 | XL_00213365 | RPL12 | 1 | 0 | 1 | 8 |
| Cdk1 | XL_00264918 | TPR | 1 | 0 | 0 | 7 |
| Cdk2 | XL_00264918 | TPR | 1 | 0 | 1 | 7 |
| Cdk3 | XL_00264918 | TPR | 1 | 0 | 0 | 7 |
| Plk3 | XL_00237045 | GBF1 | 1 | 0 | 0 | 7 |
| Plk3 | XL_00185874 | RNF113A | 0 | 1 | 0 | 7 |
| Plk3 | XL_00220553 | LIG1 | 1 | 0 | 0 | 7 |
| Cdk1 | XL_00220553 | LIG1 | 1 | 0 | 1 | 7 |
| Cdk2 | XL_00220553 | LIG1 | 1 | 0 | 1 | 7 |
| Chk1 | XL_00250393 | PDPK1 | 0 | 0 | 0 | 7 |
| Plk1 | XL_00260667 |  | 0 | 0 | 0 | 7 |
| Cdk1 | XL_00257718 |  | 1 | 0 | 0 | 7 |
| Cdk1 | XL_00274377 | NUP35 | 1 | 0 | 0 | 7 |
| Cdk2 | XL_00257718 |  | 1 | 0 | 0 | 7 |
| Cdk2 | XL_00274377 |  | 1 | 0 | 0 | 7 |
| Akt | XL_00040318 | LEO1 | 0 | 0 | 0 | 7 |
| Akt | XL_00176313 | PUM2 | 1 | 0 | 0 | 7 |
| Plk3 | XL_00069300 | NUP98 | 1 | 0 | 0 | 7 |
| Cdk1 | XL_00069300 | NUP98 | 1 | 0 | 1 | 7 |
| Cdk2 | XL_00069300 | NUP98 | 1 | 0 | 1 | 7 |
| Cdk3 | XL_00069300 | NUP98 | 1 | 0 | 0 | 7 |
| Cdk1 | XL_00037180 | SUPT5H | 0 | 1 | 1 | 7 |
| Cdk2 | XL_00037180 | SUPT5H | 0 | 1 | 1 | 7 |
| Cdk3 | XL_00037180 | SUPT5H | 0 | 1 | 0 | 7 |
| Nek6 | XL_00277348 | EEF1B2 | 0 | 0 | 0 | 7 |
| Plk3 | XL_00289633 | EIF5B | 1 | 0 | 0 | 7 |
| Cdk3 | XL_00213365 | RPL12 | 1 | 0 | 0 | 7 |
| Nek6 | XL_00213365 | RPL12 | 1 | 0 | 0 | 7 |
| Plk1 | XL_00212491 |  | 0 | 0 | 0 | 7 |
| Plk3 | XL_00212491 |  | 0 | 0 | 0 | 7 |
| Chk1 | XL_00212491 |  | 0 | 0 | 0 | 7 |
| Cdk1 | XL_00212491 |  | 0 | 0 | 0 | 7 |
| Cdk2 | XL_00212491 |  | 0 | 0 | 0 | 7 |
| Akt | XL_00212491 |  | 0 | 0 | 0 | 7 |
| Cdk1 | XL_00143764 | CAD | 0 | 0 | 0 | 7 |
| Cdk2 | XL_00143764 | CAD | 0 | 0 | 0 | 7 |
| Cdk3 | XL_00143764 | CAD | 0 | 0 | 0 | 7 |
| Plk3 | XL_00086777 | AP3D1 | 0 | 0 | 0 | 7 |
