# Supplementary material for: Prediction of Functionally Important Phospho-Regulatory Events in Xenopus laevis Oocytes
Source: PLoS Comput Biol. 2015 Aug 27;11(8):e1004362. doi: 10.1371/journal.pcbi.1004362 (PMC4552029; doi:10.1371/journal.pcbi.1004362)
Supplement: S5 Table — (DOC) [file pcbi.1004362.s009.doc]

Supplementary Table 5 – Number of phosphopeptides identified per HILIC fraction.

| HILIC Fraction # | HILIC Retention Time (min) | Mitotis 1 # Phosphopeptides | Mitosis 2 # Phosphopeptides | Interphase 1 # Phosphopeptides | Interphase 2 # Phosphopeptides |
| --- | --- | --- | --- | --- | --- |
| 1 | 4-7 | 0 | 1 | 0 | 0 |
| 2 | 12-15 | 4 | 8 | 0 | 0 |
| 3 | 16-19 | 1 | 11 | 0 | 0 |
| 4 | 20-23 | 19 | 3 | 0 | 0 |
| 5 | 24-27 | 40 | 23 | 28 | 42 |
| 6 | 29-31 | 160 | 60 | 82 | 124 |
| 7 | 32-35 | 315 | 214 | 75 | 106 |
| 8 | 36-39 | 302 | 321 | 121 | 154 |
| 9 | 40-43 | 228 | 294 | 86 | 100 |
| 10 | 44-47 | 130 | 240 | 69 | 75 |
| 11 | 48-51 | 100 | 158 | 23 | 30 |
| 12 | 52-54 | 39 | 99 | 8 | 18 |
| 13 | 55-56 | 37 | 34 | 15 | 33 |
